# Development and external validation of an explainable XGBoost model with a web-based calculator for risk prediction of postpartum depressive symptoms

**DOI:** 10.3389/fpsyt.2026.1887667

**Published:** 2026-09-15

**Authors:** Qian Zhang, Fang Wu, Mei Zhu, Liwei Sun

**Affiliations:** 1 Department of Obstetrics and Gynecology, the Second People’s Hospital of Bengbu City, Bengbu, China; 2 Department of Obstetrics and Gynecology, General Hospital of Huainan Oriental Hospital Group, Huainan, China

**Keywords:** machine learning, model interpretability, postpartum depression, web-based calculator, XGBoost

## Abstract

**Introduction:**

Postpartum depression (PPD) is associated with severe adverse outcomes, yet timely risk stratification remains challenging. Existing machine learning models are often limited by insufficient external validation, limited interpretability, and lack of accessible clinical tools. This study aimed to develop and externally validate an explainable machine learning model with a web-based calculator for predicting postpartum depressive symptoms.

**Methods:**

This retrospective cohort study included 553 women from two hospitals, comprising 403 in the development cohort and 150 in an independent external validation cohort. The development cohort was divided into training and internal test sets. Screening-positive postpartum depressive symptoms at 6 weeks postpartum were defined as an Edinburgh Postnatal Depression Scale (EPDS) score ≥9. Five machine learning models were developed using demographic, psychosocial, and obstetric predictors. Model discrimination was assessed using the area under the receiver operating characteristic curve (AUC), with calibration and decision curve analyses performed additionally. SHapley Additive exPlanations (SHAP) were used for model interpretation.

**Results:**

Screening-positive rates were 38.0% in the development cohort and 26.0% in the external validation cohort. Tree-based ensemble models showed broadly comparable performance. eXtreme Gradient Boosting (XGBoost) was selected based on its numerically highest mean cross-validated AUC and balanced overall performance, with AUCs of 0.895 ± 0.033 in cross-validation, 0.845 (95% confidence interval [CI]: 0.751–0.940) in the internal test set, and 0.820 (95% CI: 0.743–0.889) in the external validation cohort. Calibration was acceptable in the internal test set, whereas absolute risk was overestimated in the external validation cohort. Decision curve analysis indicated potential net clinical benefit. SHAP identified age, marital relationship quality, prenatal depressive symptoms, monthly personal income, and sleep disturbance as important model-based contributors. A four-predictor web calculator was developed.

**Discussion:**

The explainable XGBoost model showed promising discrimination for predicting EPDS-defined postpartum depressive symptoms. However, given the modest external validation sample and evidence of risk overestimation, further prospective multicenter validation and recalibration are needed before routine clinical implementation.

## 1 Introduction

Postpartum depression (PPD) is one of the most prevalent psychiatric disorders during the perinatal period and is characterized by persistent low mood, anhedonia, and diminished interest in daily activities (1). A large-scale meta-analysis involving 296,284 postpartum women across 56 countries reported a pooled global prevalence of 17.7%, with rates reaching approximately 38% in some regions (2–4). Recent social stressors, including the COVID-19 pandemic, may have further increased this burden (5, 6). The adverse consequences of PPD affect both mothers and their offspring. In mothers, PPD is associated with an increased risk of self-harm and suicidal ideation and, in severe cases, infanticide (7–9). In offspring, maternal PPD has been associated with increased vulnerability to psychiatric disorders and delayed cognitive development later in life (10–14). PPD therefore represents an important global public health concern, with substantial consequences for individual health, family well-being, and healthcare systems (15). Developing practical risk prediction tools to identify women at elevated risk may facilitate timely assessment and targeted management.

Machine learning (ML) provides flexible approaches for integrating multidimensional and potentially complex data and has increasingly been applied to clinical prediction research (16, 17). Previous studies suggest that postpartum depressive symptoms are associated with a range of demographic, physiological, psychological, social, and obstetric factors (18–20). Integrating these multidimensional predictors may therefore facilitate individualized risk estimation and early screening (21, 22). Although several ML-based prediction models for PPD have shown promising performance (23), important limitations remain. Some studies have relied on limited feature selection or modeling strategies (22, 24, 25), while others have lacked independent external validation, limiting assessment of model transportability across populations and clinical settings (26, 27). In addition, relatively few studies have translated prediction models into accessible tools for individualized risk estimation (28, 29).

Accordingly, this study aimed to integrate multidimensional clinical data and compare multiple machine learning algorithms to develop a model for predicting postpartum depressive symptoms. The selected model was evaluated using both a held-out internal test set and an independent external validation cohort. Model interpretability was further assessed using SHAP, and a simplified web-based calculator was developed to facilitate individualized risk estimation.

## 2 Materials and methods

### 2.1 Study participants

To reduce potential selection bias, consecutive perinatal women who received routine antenatal care, delivered, and underwent postpartum follow-up at two medical centers between 2023 and 2025 were screened according to predefined eligibility criteria. A total of 613 women were initially screened, including 449 from the Second People’s Hospital of Bengbu City (SPHBC) for the development cohort and 164 from the General Hospital of Huainan Oriental Hospital Group (GHHOHG) for the independent external validation cohort. After excluding 60 women, including 10 who declined participation, 45 with substantially incomplete baseline or predictor data, and 5 who were lost to follow-up at 6 weeks postpartum, 553 women were included in the final analysis, comprising 403 in the development cohort and 150 in the independent external validation cohort. Using outcome-stratified random sampling with a fixed random seed of 417, the development cohort was divided at an 8:2 ratio into a training set (n = 322) and a held-out internal test set (n = 81). The independent external validation cohort was kept separate throughout model development. **Figure 1** illustrates participant enrollment, data partitioning, and the complete machine learning workflow. This study was approved by the Ethics Committee of SPHBC (Approval No. 2025-02) and the Ethics Committee of GHHOHG (Approval No. DF-LL-KY-2026-03). Written informed consent was obtained from all participants or their legal guardians.

**Figure 1:**
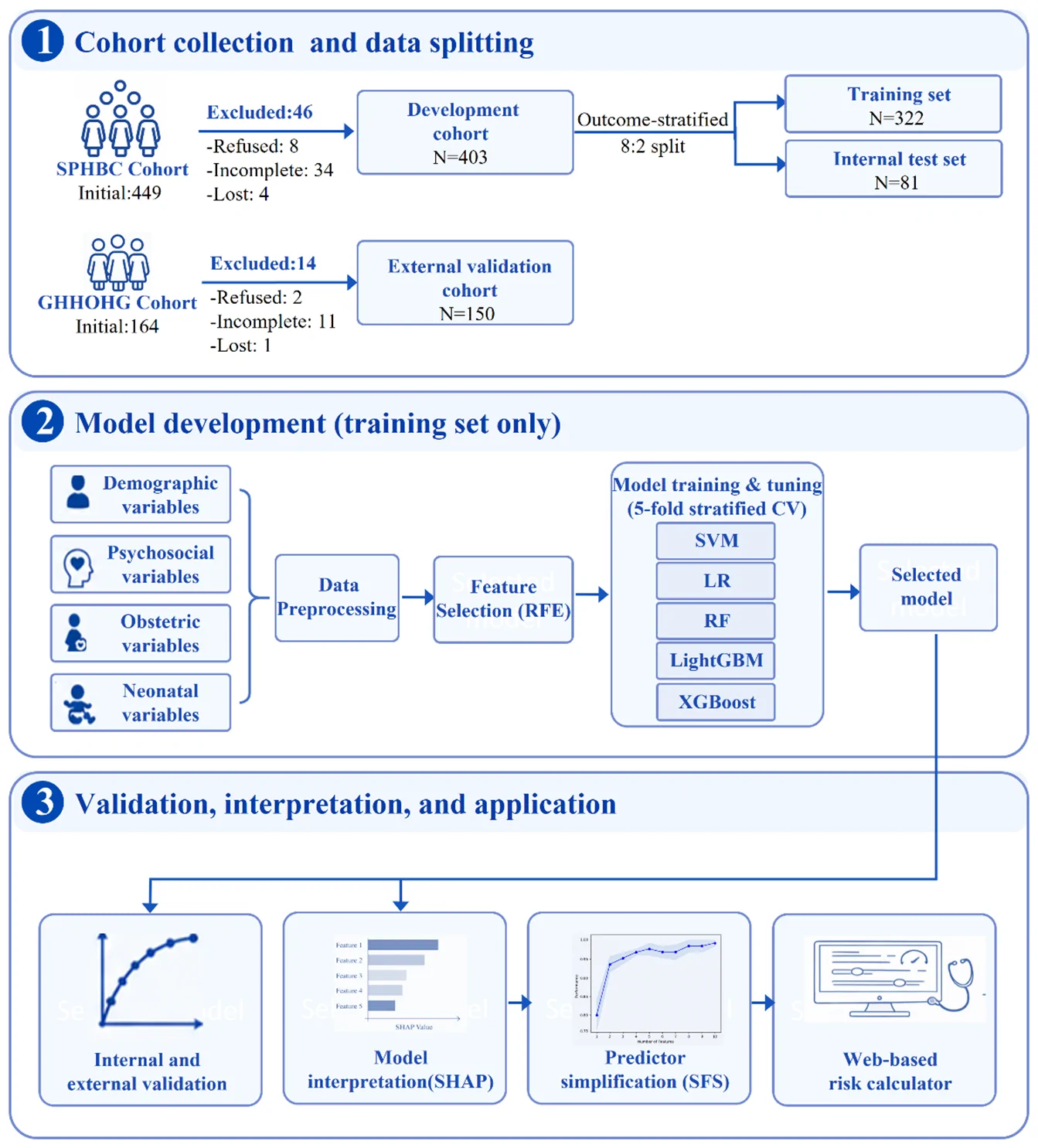
Flowchart of the participant enrollment and study design. The development cohort was divided into a training set and a held-out internal test set using outcome-stratified random sampling. Model development, including preprocessing, RFE, and five-fold stratified cross-validation for model training and tuning, was restricted to the training set. The selected model was evaluated in the internal test set and independent external validation cohort, followed by SHAP interpretation, SFS-based predictor simplification, and web-based calculator development. SPHBC, the Second People’s Hospital of Bengbu City; GHHOHG, General Hospital of Huainan Oriental Hospital Group; RFE, recursive feature elimination; CV, cross-validation; SVM, support vector machine; LR, logistic regression; RF, random forest; LightGBM, Light Gradient Boosting Machine; XGBoost, eXtreme Gradient Boosting; SHAP, SHapley Additive exPlanations; SFS, sequential forward selection.

### 2.2 Data collection and predictor definitions

Informed by previous literature, candidate predictors were collected across demographic, obstetric, neonatal, and psychosocial domains (25, 26, 30). To clarify the temporal relationship between predictors and the outcome, all candidate predictors were available before the 6-week postpartum outcome assessment. Demographic variables, including age, years of education, and monthly personal income, were obtained using structured questionnaires at enrollment during delivery hospitalization. Obstetric and neonatal variables, including gravidity, parity, gestational age, mode of delivery, infant feeding method, neonatal health status, gestational hypertension, and gestational diabetes mellitus, were extracted from electronic medical records before or at postpartum discharge. Consistent with established approaches in perinatal prediction studies, psychosocial predictors were assessed using structured self-report questionnaire items during hospitalization or postpartum follow-up before the outcome assessment (31, 32). Prenatal depressive symptoms were defined as self-reported depressive symptoms during pregnancy and coded as present or absent. Postpartum negative life events were defined as self-reported negative or stressful life events occurring after delivery and before the 6-week postpartum assessment and coded as yes or no. Sleep disturbance was defined as self-reported sleep-related problems during the perinatal period and coded as yes or no. Marital relationship quality was assessed according to participants’ self-reported overall marital relationship and categorized as good or poor. Perceived living conditions were evaluated based on satisfaction with the current living environment and categorized as satisfied or dissatisfied. These psychosocial variables were used as candidate predictors rather than diagnostic measures, and no variables measured after the outcome assessment were used for model development.

### 2.3 Outcome definition

The primary outcome was screening-positive postpartum depressive symptoms assessed at 6 weeks postpartum using the Chinese version of the Edinburgh Postnatal Depression Scale (EPDS) (28). The EPDS consists of 10 items, each scored from 0 to 3, yielding a total score ranging from 0 to 30, with higher scores indicating more severe depressive symptoms. In this study, an EPDS score ≥9 was used to define a positive screen for postpartum depressive symptoms. This threshold was selected to prioritize early risk identification and reduce missed cases in the context of postpartum screening, consistent with previous studies that applied this threshold for early postpartum screening (29, 33). Because no structured clinical psychiatric interview was conducted, an EPDS score ≥9 should be interpreted as indicating screening-positive postpartum depressive symptoms rather than a definitive clinical diagnosis of postpartum depression. For clarity and brevity, participants with EPDS scores ≥9 were classified as the EPDS-positive group, whereas those with EPDS scores <9 were classified as the EPDS-negative group.

### 2.4 Data preprocessing and feature selection

Missingness was assessed for all candidate predictors and the 6-week EPDS outcome. As no missing values were identified among the 553 participants included in the final analysis, no missing-data imputation was performed. All preprocessing procedures were performed after the development cohort had been divided into the training and held-out internal test sets. Continuous variables were standardized using Z-score transformation, binary categorical variables were coded as 0 or 1, and monthly personal income, which comprised three categories, was transformed using one-hot encoding.

Recursive feature elimination (RFE) was used for feature selection by iteratively removing the least informative predictors (34). Preprocessing and RFE were incorporated into the five-fold stratified cross-validation procedure within the training set. Within each cross-validation iteration, preprocessing parameters and feature selection were fitted using only the training folds and then applied to the corresponding validation fold without refitting. Thus, information from each validation fold was not used to estimate preprocessing parameters or select predictors. After model development, the final preprocessing procedure and predictor set were determined using the full training set and were subsequently applied without refitting to the held-out internal test set and independent external validation cohort.

### 2.5 Machine learning models

Five machine learning algorithms were developed and systematically compared: support vector machine (SVM), logistic regression (LR), random forest (RF), Light Gradient Boosting Machine (LightGBM), and eXtreme Gradient Boosting (XGBoost). SVM identifies a separating hyperplane by maximizing the margin between classes, whereas LR provides an interpretable linear probabilistic model. RF improves predictive robustness by aggregating multiple decision trees constructed from bootstrap samples and randomly selected predictors. LightGBM and XGBoost are gradient-boosting tree algorithms capable of capturing nonlinear relationships and interactions, with LightGBM emphasizing computational efficiency and XGBoost incorporating regularization to reduce overfitting.

Hyperparameter optimization was performed exclusively within the training set using randomized search with five-fold stratified cross-validation, with the mean area under the receiver operating characteristic curve (AUC) used as the optimization criterion and a fixed random seed of 417. For each sampled hyperparameter configuration, the model was fitted using four folds and evaluated on the remaining fold, with each fold serving once as the validation fold. Preprocessing and RFE were fitted only within the training folds and applied to the corresponding validation folds without refitting. The hyperparameter search spaces and final selected values for all five models are provided in **Supplementary Table 1**.

After hyperparameter tuning, each model was refitted using the entire training set with the selected hyperparameters. The final model was selected according to its cross-validated performance in the training set and was subsequently evaluated in the held-out internal test set. For comparative purposes, all five candidate models were additionally evaluated in the independent external validation cohort. Neither the held-out internal test set nor the external validation cohort was used for preprocessing parameter estimation, feature selection, hyperparameter tuning, or model selection. The EPDS screening outcome was used only as the supervised learning label and was not included as a predictor.

Predictive performance was evaluated using AUC, accuracy, sensitivity, specificity, precision, and F1 score. For external validation, 95% confidence intervals (CIs) for AUC, accuracy, sensitivity, specificity, precision, and F1 score were estimated using 1,000 bootstrap resamples. Given the observed class distribution, no class weighting or resampling techniques were applied, and the original outcome distribution was retained. Stratified data splitting and cross-validation were used to preserve class proportions. A fixed probability threshold of 0.50 was used to derive binary classifications and was applied unchanged to the held-out internal test set and independent external validation cohort. The threshold was not re-optimized in either validation dataset. For the final selected model, calibration was evaluated in both validation datasets using calibration plots, calibration intercepts, calibration slopes, and Brier scores. Decision curve analysis was performed in both validation datasets to evaluate net benefit across a range of threshold probabilities relative to the treat-all and treat-none strategies. Confusion matrices were also generated for the final selected model in both validation datasets.

### 2.6 Model interpretation and web-based calculator development

SHapley Additive exPlanations (SHAP) were applied to the final XGBoost model fitted using the training set to quantify the contribution of each predictor to individual predictions and assess overall feature importance. To develop a more parsimonious model, SHAP-based feature importance was combined with sequential forward selection (SFS) (17). Candidate predictor subsets were evaluated exclusively within the training set using five-fold stratified cross-validation and AUC. Neither the held-out internal test set nor the independent external validation cohort was used to calculate SHAP-based feature importance or select the simplified predictor subset. The simplified predictor set was fixed before development of the web-based calculator. This predictor-simplification procedure did not influence the selection or reported validation performance of the full XGBoost model.

### 2.7 Statistical analysis

Statistical analyses were conducted using R version 4.4.1 and Python version 3.11.4. The Python-based machine learning workflow included NumPy version 1.23.5, pandas version 2.0.3, SciPy version 1.11.1, and scikit-learn version 1.2.2, with XGBoost version 1.7.4 and LightGBM version 3.3.5 used for gradient-boosting model development. Model interpretation was conducted using SHAP version 0.41.0, and figures were generated using Matplotlib version 3.7.1 and Seaborn version 0.12.2. Categorical variables were presented as frequencies and percentages and compared using the χ² test or Fisher’s exact test. Continuous variables were expressed as mean ± standard deviation or median with interquartile range, depending on distribution, and compared using the Student’s t-test or nonparametric tests as appropriate. All statistical tests were two-sided, and a P-value <0.05 was considered statistically significant. This study was reported in accordance with the Transparent Reporting of a multivariable prediction model for Individual Prognosis Or Diagnosis plus Artificial Intelligence (TRIPOD+AI) reporting guideline (35).

## 3 Results

### 3.1 Comparison of baseline characteristics

**Table 1** summarizes the baseline characteristics of postpartum women in the development cohort and independent external validation cohort. In total, 553 postpartum women were enrolled, including 403 in the development cohort and 150 in the external validation cohort. Generally, no statistically significant differences were observed between the two cohorts in most clinical and sociodemographic characteristics, including age, neonatal health status, infant feeding method, gestational hypertension, gestational diabetes mellitus, marital relationship quality, postpartum negative life events, prenatal depressive symptoms, monthly personal income, and sleep disturbance (all *P* > 0.05). However, significant differences were observed in several variables. Compared with the development cohort, postpartum women in the external validation cohort had significantly higher years of education (13.97 ± 2.76 vs. 12.21 ± 3.57 years, *P* < 0.001), whereas those in the development cohort had significantly lower gravidity (2.68 ± 1.49 vs. 1.91 ± 1.07, *P* < 0.001). Regarding delivery-related characteristics, the proportion of cesarean section was significantly higher in the external validation cohort than in the development cohort (64.0% vs. 42.2%, *P* < 0.001). Similarly, the proportion of primiparous women was also significantly greater in the external validation cohort (59.3% vs. 38.2%, *P* < 0.001). Additionally, the incidence of preterm birth was higher in the development cohort than in the external validation cohort (6.2% vs. 0.0%, *P* = 0.002). Conversely, the proportion of participants reporting unsatisfactory living conditions was greater in the external validation cohort (51.3% vs. 36.7%, χ² = 9.67, *P* = 0.002). A statistically significant difference in the proportion of EPDS-defined screening-positive postpartum depressive symptoms was also observed between the two cohorts. The screening-positive rate was higher in the development cohort than in the external validation cohort (38.0% vs. 26.0%, χ² = 6.905, *P* = 0.009).

**Table 1:** Comparison of demographic and clinical characteristics between the development cohort and the external validation cohort.

Characteristic	All data (n=553)	Development cohort (n=403)	External validation cohort (n=150)	*t*/Z/χ²	*P*-value
Age, y	29.95 ± 4.69	30.05 ± 4.89	29.68 ± 4.02	0.824	0.410
Education, y	12.69 ± 3.46	12.21 ± 3.57	13.97 ± 2.76	-5.465	< 0.001
Gravidity, n	2.47 ± 1.43	2.68 ± 1.49	1.91 ± 1.07	5.853	< 0.001
Mode of delivery				20.841	< 0.001
Vaginal delivery	287 (51.9%)	233 (57.8%)	54 (36.0%)		
Cesarean section	266 (48.1%)	170 (42.2%)	96 (64.0%)		
Parity				19.794	< 0.001
Primiparous	243 (43.9%)	154 (38.2%)	89 (59.3%)		
Multiparous	310 (56.1%)	249 (61.8%)	61 (40.7%)		
Neonatal health status				0.423	0.515
Good	548 (99.1%)	400 (99.3%)	148 (98.7%)		
Poor	5 (0.9%)	3 (0.7%)	2 (1.3%)		
Gestational age				9.746	0.002
Full-term	528 (95.5%)	378 (93.8%)	150 (100.0%)		
Preterm	25 (4.5%)	25 (6.2%)	0 (0.0%)		
Infant feeding method				0.027	0.869
Breastfeeding	451 (81.6%)	328 (81.4%)	123 (82.0%)		
Formula feeding	102 (18.4%)	75 (18.6%)	27 (18.0%)		
Gestational hypertension				2.093	0.148
No	536 (96.9%)	388 (96.3%)	148 (98.7%)		
Yes	17 (3.1%)	15 (3.7%)	2 (1.3%)		
Gestational diabetes mellitus				3.021	0.082
No	545 (98.6%)	395 (98.0%)	150 (100.0%)		
Yes	8 (1.4%)	8 (2.0%)	0 (0.0%)		
Perceived living conditions				9.667	0.002
Satisfied	328 (59.3%)	255 (63.3%)	73 (48.7%)		
Dissatisfied	225 (40.7%)	148 (36.7%)	77 (51.3%)		
Marital relationship quality				0.028	0.868
Good	423 (76.5%)	309 (76.7%)	114 (76.0%)		
Poor	130 (23.5%)	94 (23.3%)	36 (24.0%)		
Postpartum negative life events				0.636	0.425
No	384 (69.4%)	276 (68.5%)	108 (72.0%)		
Yes	169 (30.6%)	127 (31.5%)	42 (28.0%)		
Prenatal depressive symptoms				0.471	0.493
No	356 (64.4%)	256 (63.5%)	100 (66.7%)		
Yes	197 (35.6%)	147 (36.5%)	50 (33.3%)		
Monthly personal income, CNY				0.445	0.801
< 5,000	215 (38.9%)	160 (39.7%)	55 (36.7%)		
5,000-10,000	215 (38.9%)	154 (38.2%)	61 (40.7%)		
> 10,000	123 (22.2%)	89 (22.1%)	34 (22.7%)		
Sleep disturbance				2.171	0.141
No	311 (56.2%)	219 (54.3%)	92 (61.3%)		
Yes	242 (43.8%)	184 (45.7%)	58 (38.7%)		
Outcome				6.905	0.009
No	361 (65.3%)	250 (62.0%)	111 (74.0%)		
Yes	192 (34.7%)	153 (38.0%)	39 (26.0%)		

CNY, Chinese Yuan.

**Table 2** presents the baseline characteristics of postpartum women according to EPDS screening status in the development cohort. Among the 403 postpartum women in the development cohort, 153 screened positive for postpartum depressive symptoms, while 250 screened negative. Compared with the EPDS-negative group, women in the EPDS-positive group were significantly younger (28.46 ± 5.77 vs. 31.02 ± 3.97 years, *P* < 0.001) and had a significantly lower number of pregnancies (2.37 ± 1.46 vs. 2.87 ± 1.47, *P* = 0.001). The proportion of cesarean delivery was significantly higher in the EPDS-positive group than in the EPDS-negative group (55.6% vs. 34.0%, *P* < 0.001). In addition, women in the EPDS-positive group were more likely to report unsatisfactory living conditions (57.5% vs. 24.0%, *P* < 0.001) and disharmonious marital relationships compared with those in the EPDS-negative group (42.5% vs. 11.6%, *P* < 0.001). The EPDS-positive group also exhibited a higher incidence of negative postpartum life events (41.8% vs. 25.2%, P < 0.001) and a significantly greater prevalence of prenatal depressive symptoms (61.4% vs. 21.2%, *P* < 0.001). The proportion of women with low monthly personal income was also significantly higher in the EPDS-positive group compared with the EPDS-negative group (*P* < 0.001). Furthermore, sleep disturbance was more common in the EPDS-positive group (63.4% vs. 34.8%, *P* < 0.001). In contrast, no statistically significant differences were observed between the two groups in years of education, parity, neonatal health status, gestational age, infant feeding method, gestational hypertension, or gestational diabetes mellitus (all *P* > 0.05).

**Table 2:** Comparison of baseline characteristics according to EPDS screening status in the development cohort.

Characteristic	All data (n=403)	EPDS-positive group (n=153)	EPDS-negative group (n=250)	*t*/Z/χ²	*P*-value
Age, y	30.05 ± 4.89	28.46 ± 5.77	31.02 ± 3.97	-5.284	< 0.001
Education, y	12.21 ± 3.57	11.99 ± 3.66	12.35 ± 3.52	-0.984	0.326
Gravidity, n	2.68 ± 1.49	2.37 ± 1.46	2.87 ± 1.47	-3.136	0.001
Mode of delivery				18.082	< 0.001
Vaginal delivery	233 (57.8%)	68 (44.4%)	165 (66.0%)		
Cesarean section	170 (42.2%)	85 (55.6%)	85 (34.0%)		
Parity				0.096	0.757
Primiparous	154 (38.2%)	57 (37.3%)	97 (38.8%)		
Multiparous	249 (61.8%)	96 (62.7%)	153 (61.2%)		
Neonatal health status				1.057	0.304
Good	400 (99.3%)	151 (98.7%)	249 (99.6%)		
Poor	3 (0.7%)	2 (1.3%)	1 (0.4%)		
Gestational age				0.047	0.829
Full-term	378 (93.8%)	143 (93.5%)	235 (94.0%)		
Preterm	25 (6.2%)	10 (6.5%)	15 (6.0%)		
Infant feeding method				2.124	0.145
Breastfeeding	328 (81.4%)	119 (77.8%)	209 (83.6%)		
Formula feeding	75 (18.6%)	34 (22.2%)	41 (16.4%)		
Gestational hypertension				3.212	0.073
No	388 (96.3%)	144 (94.1%)	244 (97.6%)		
Yes	15 (3.7%)	9 (5.9%)	6 (2.4%)		
Gestational diabetes mellitus				0.583	0.445
No	395 (98.0%)	151 (98.7%)	244 (97.6%)		
Yes	8 (2.0%)	2 (1.3%)	6 (2.4%)		
Perceived living conditions				45.883	< 0.001
Satisfied	255 (63.3%)	65 (42.5%)	190 (76.0%)		
Dissatisfied	148 (36.7%)	88 (57.5%)	60 (24.0%)		
Marital relationship quality				50.618	< 0.001
Good	309 (76.7%)	88 (57.5%)	221 (88.4%)		
Poor	94 (23.3%)	65 (42.5%)	29 (11.6%)		
Postpartum negative life events				12.162	< 0.001
No	276 (68.5%)	89 (58.2%)	187 (74.8%)		
Yes	127 (31.5%)	64 (41.8%)	63 (25.2%)		
Prenatal depressive symptoms				66.321	< 0.001
No	256 (63.5%)	59 (38.6%)	197 (78.8%)		
Yes	147 (36.5%)	94 (61.4%)	53 (21.2%)		
Monthly personal income, CNY				58.177	< 0.001
< 5,000	160 (39.7%)	78 (51.0%)	82 (32.8%)		
5,000-10,000	154 (38.2%)	72 (47.1%)	82 (32.8%)		
> 10,000	89 (22.2%)	3 (2.0%)	86 (34.4%)		
Sleep disturbance				31.287	< 0.001
No	219 (54.3%)	56 (36.6%)	163 (65.2%)		
Yes	184 (45.7%)	97 (63.4%)	87 (34.8%)		

EPDS-positive was defined as an EPDS score ≥9, whereas EPDS-negative was defined as an EPDS score <9.

CNY, Chinese Yuan.

### 3.2 Results of feature selection

To minimize feature redundancy and improve predictive stability, RFE was employed for candidate variable selection. Based on this method, the final set of selected predictive features included age, marital relationship quality, prenatal depressive symptoms, monthly personal income, sleep disturbance, mode of delivery, gestational age, parity, perceived living conditions, postpartum negative life events, years of education, and infant feeding method.

### 3.3 Performance of multiple machine learning models

In the training set, five-fold stratified cross-validation was used to compare the predictive performance of LR, SVM, RF, XGBoost, and LightGBM. The mean AUC values were 0.862 ± 0.020 for LR, 0.860 ± 0.019 for SVM, 0.891 ± 0.027 for RF, 0.895 ± 0.033 for XGBoost, and 0.894 ± 0.035 for LightGBM (**Figure 2**). Detailed cross-validated performance metrics are presented in **Table 3**. The three tree-based ensemble models showed broadly comparable performance, and no formal pairwise statistical comparisons were performed. XGBoost was selected as the final model based on its numerically highest mean cross-validated AUC and balanced overall performance. In the held-out internal test set, the selected XGBoost model achieved an AUC of 0.845 (95% CI: 0.751–0.940) (**Figure 3A**). External validation results with 95% confidence intervals for all five candidate models are presented in **Table 4**. AUC values ranged from 0.800 to 0.820, indicating broadly comparable discrimination across models. XGBoost achieved an AUC of 0.820 (95% CI: 0.743–0.889) in the independent external validation cohort (**Figure 3B**). Confusion matrices for the XGBoost model at the fixed probability threshold of 0.50 are shown in **Supplementary Figure 1**.

**Figure 2:**
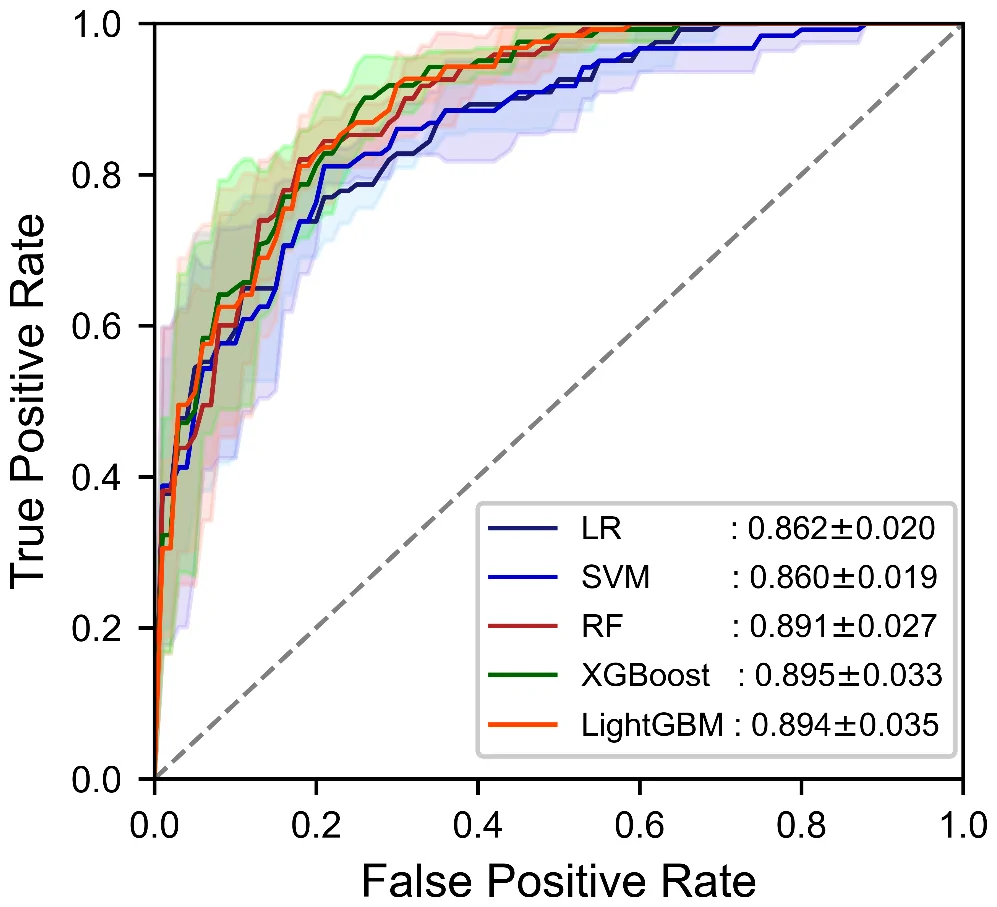
The average AUC performance of five machine learning models subjected to fivefold cross-validation. The figure shows the mean ROC curves and corresponding AUC values from five-fold cross-validation for LR, SVM, RF, XGBoost, and LightGBM. Shaded areas indicate variability across validation folds. AUC, area under the receiver operating characteristic curve; LightGBM, Light Gradient Boosting Machine; LR, logistic regression; RF, random forest; ROC, receiver operating characteristic; SVM, support vector machine; XGBoost, eXtreme Gradient Boosting.

**Table 3:** Comparative performance of five machine learning models based on five-fold cross-validation in the training set.

Models	AUC	Accuracy	Sensitivity	Specificity	Precision	F1 score
LR	0.862	0.814	0.704	0.880	0.795	0.742
SVM	0.860	0.814	0.713	0.875	0.790	0.745
RF	0.891	0.823	0.704	0.895	0.806	0.749
XGBoost	0.895	0.829	0.737	0.885	0.799	0.765
LightGBM	0.894	0.832	0.738	0.890	0.808	0.769

AUC, Area under the receiver operating characteristic curve; LightGBM, Light gradient boosting machine; LR, Logistic regression; RF, Random forest; SVM, Support vector machine; XGBoost, Extreme gradient boosting.

**Figure 3:**
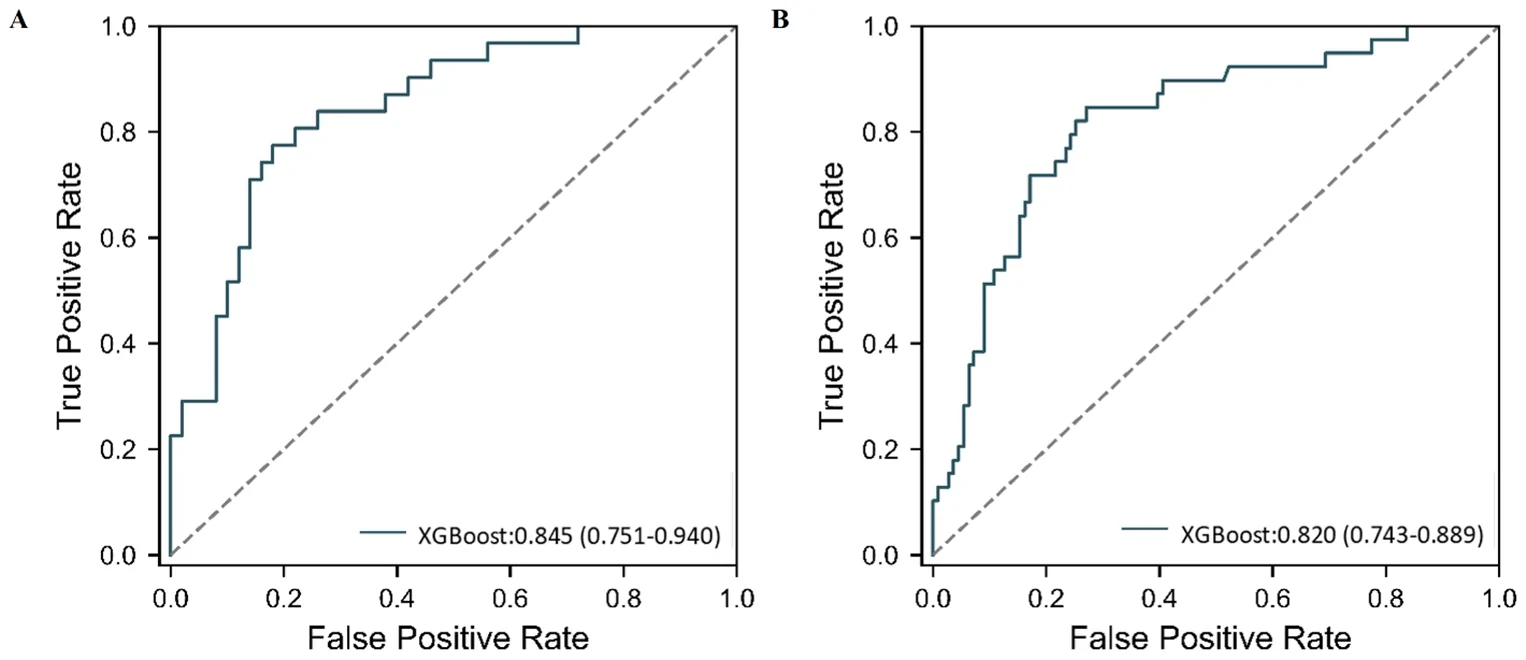
Receiver operating characteristic curves of the final XGBoost model in the internal test set and external validation cohort. **(A)** Internal test set: AUC = 0.845 (95% CI: 0.751–0.940). **(B)** External validation cohort: AUC = 0.820 (95% CI: 0.743–0.889). AUC, area under the receiver operating characteristic curve; CI, confidence interval; XGBoost, eXtreme Gradient Boosting.

**Table 4:** External validation performance of the five candidate machine learning models.

Models	AUC	Accuracy	Sensitivity	Specificity	Precision	F1 score
LR	0.817 (0.733–0.889)	0.793 (0.727–0.853)	0.692 (0.548–0.828)	0.829 (0.756–0.893)	0.587 (0.439–0.726)	0.635 (0.507–0.745)
SVM	0.818 (0.727–0.898)	0.800 (0.733–0.867)	0.769 (0.618–0.897)	0.811 (0.737–0.882)	0.588 (0.447–0.729)	0.667 (0.532–0.771)
RF	0.800 (0.706–0.879)	0.780 (0.707–0.847)	0.641 (0.500–0.784)	0.829 (0.757–0.901)	0.568 (0.423–0.722)	0.602 (0.472–0.718)
XGBoost	0.820 (0.743–0.889)	0.793 (0.720–0.853)	0.641 (0.486–0.781)	0.847 (0.778–0.903)	0.595 (0.438–0.732)	0.617 (0.474–0.720)
LightGBM	0.815 (0.739–0.887)	0.767 (0.693–0.827)	0.590 (0.432–0.744)	0.829 (0.756–0.897)	0.548 (0.400–0.692)	0.568 (0.432–0.681)

Values are presented as point estimates (95% CIs). Confidence intervals were estimated using 1,000 bootstrap resamples. AUC, Area under the receiver operating characteristic curve; LightGBM, Light gradient boosting machine; LR, Logistic regression; RF, Random forest; SVM, Support vector machine; XGBoost, Extreme gradient boosting.

Calibration of the final XGBoost model was evaluated in both validation datasets. In the internal test set, the calibration intercept was −0.036, the calibration slope was 1.052, and the Brier score was 0.155, indicating close agreement between predicted and observed risks (**Figure 4A**). In the external validation cohort, the corresponding values were −0.600, 1.021, and 0.151, respectively. Although the calibration slope remained close to 1, the negative intercept and calibration curve below the reference line indicated systematic overestimation of absolute risk in the external cohort (**Figure 4B**). Decision curve analysis showed that the final XGBoost model provided greater net benefit than the treat-all and treat-none strategies across a range of threshold probabilities in both the internal test set and external validation cohort (**Figures 4C, D**), suggesting potential clinical utility for risk stratification.

**Figure 4:**
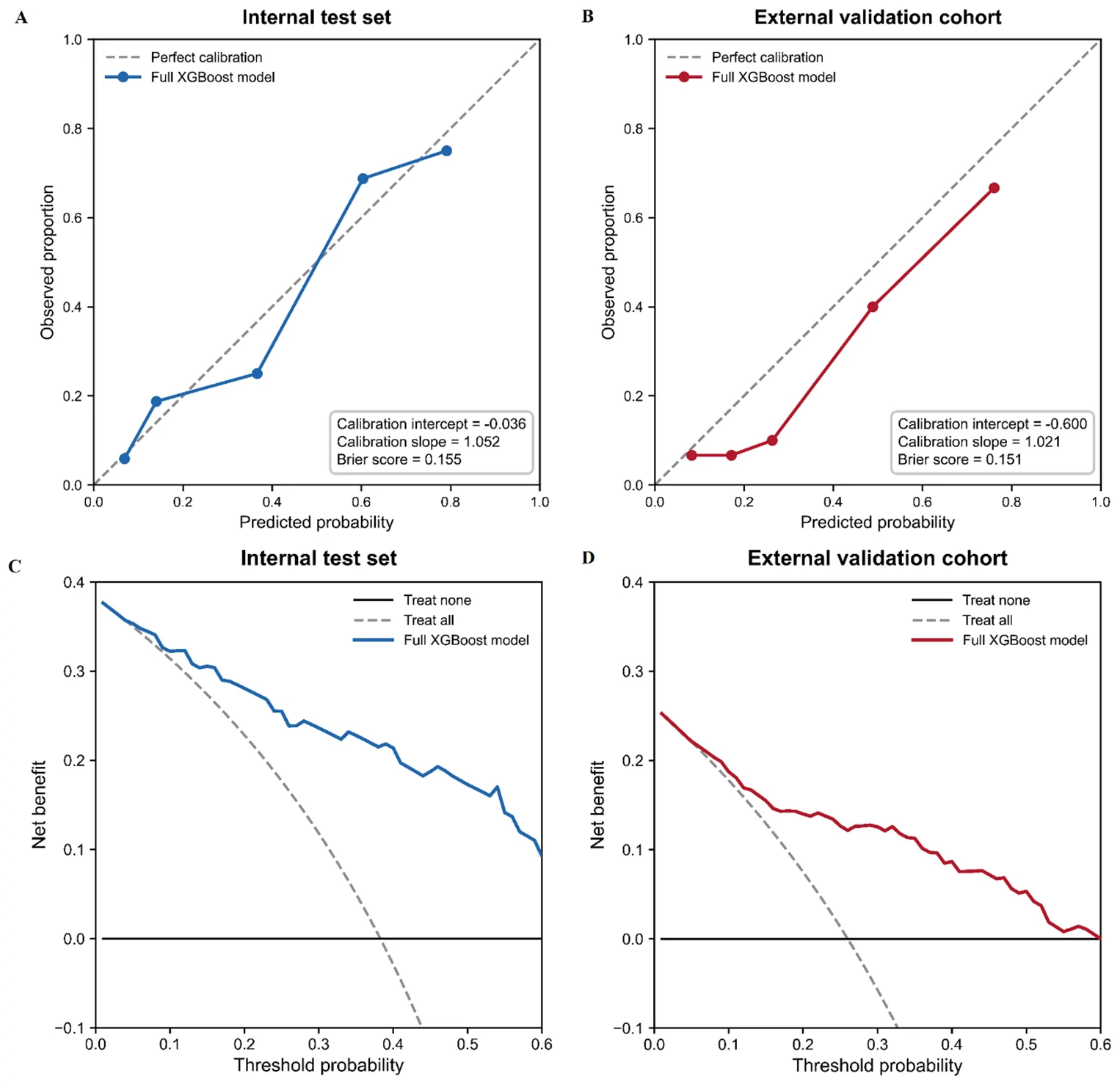
Calibration and decision curve analyses of the final XGBoost model. **(A, B)** Calibration plots for the internal test set and external validation cohort, respectively, with calibration intercepts, slopes, and Brier scores presented in the corresponding panels. **(C, D)** Decision curve analyses for the internal test set and external validation cohort, respectively, comparing the net benefit of the XGBoost model with the treat-all and treat-none strategies across a range of threshold probabilities. XGBoost, eXtreme Gradient Boosting.

### 3.4 Interpretation of the prediction model

To enhance model interpretability, SHAP analysis was applied to the XGBoost model to quantify the relative contribution of each predictor to model predictions of EPDS-defined postpartum depressive symptoms (**Figure 5**). Age was among the most influential predictors, with younger age associated with higher predicted risk within the model. Marital relationship quality was also an important contributor, with poorer marital relationships associated with higher predicted risk. Prenatal depressive symptoms and sleep disturbance were similarly associated with higher predicted risk, whereas higher monthly personal income was associated with lower predicted risk. Variables including mode of delivery, gestational age, parity, living conditions, and postpartum negative life events showed smaller overall contributions but contributed to predictions in some individuals. These SHAP findings reflect model-based associations and should not be interpreted as causal effects.

**Figure 5:**
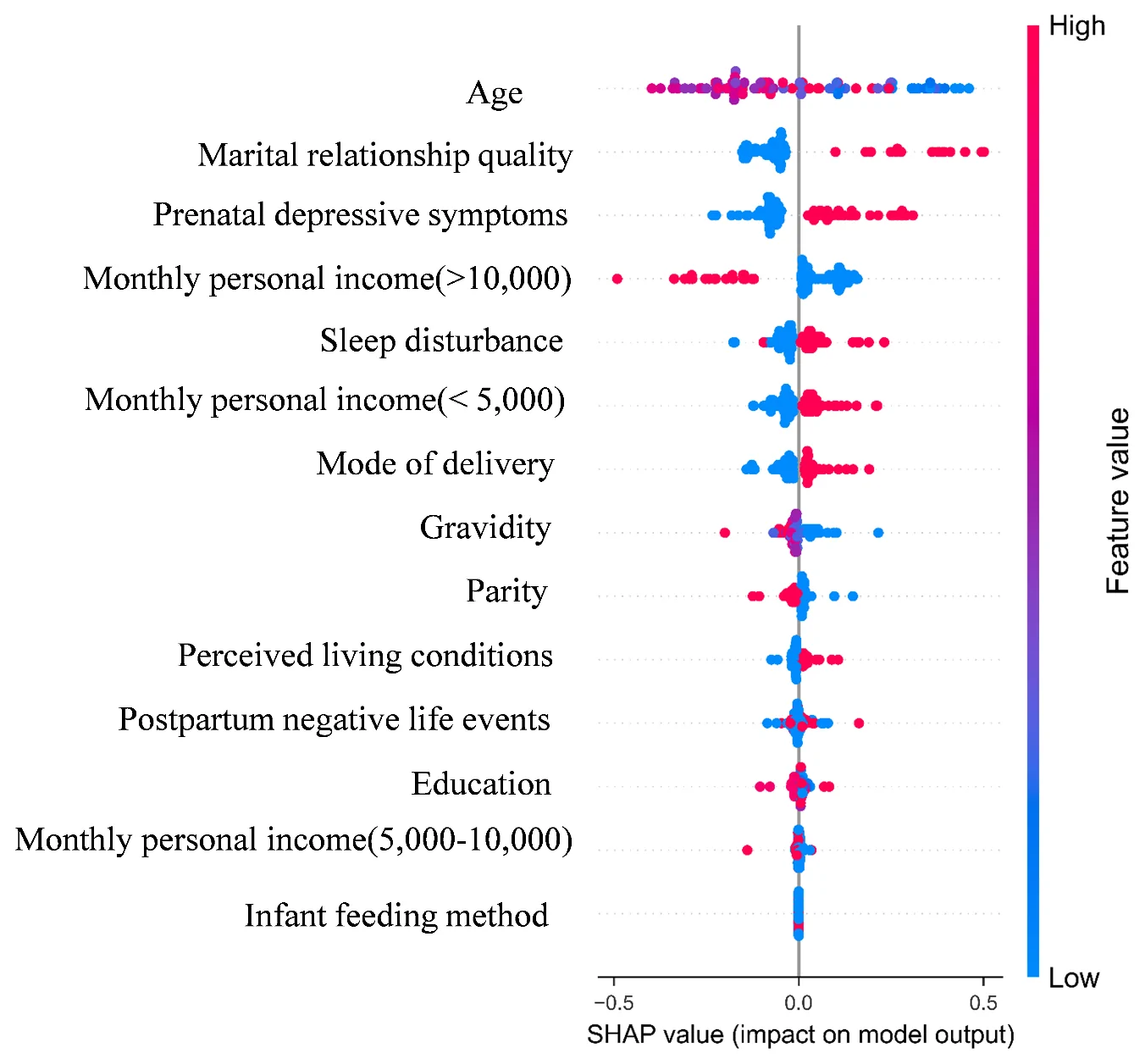
SHAP summary plot of the XGBoost model for predicting postpartum depressive symptoms. Predictors are ranked by overall importance. Each dot represents one participant, with the x-axis showing the SHAP value and color indicating the feature value from low to high. SHAP, SHapley Additive exPlanations; XGBoost, eXtreme Gradient Boosting.

### 3.5 Web-based calculator development

To develop a more parsimonious prediction tool, SHAP-based feature importance was combined with sequential forward selection (SFS). Candidate predictor subsets were sequentially evaluated according to their cross-validated discriminative performance and model parsimony. A variable importance plot and the corresponding AUC trend curve were generated to illustrate model performance across different predictor combinations (**Figure 6**).

**Figure 6:**
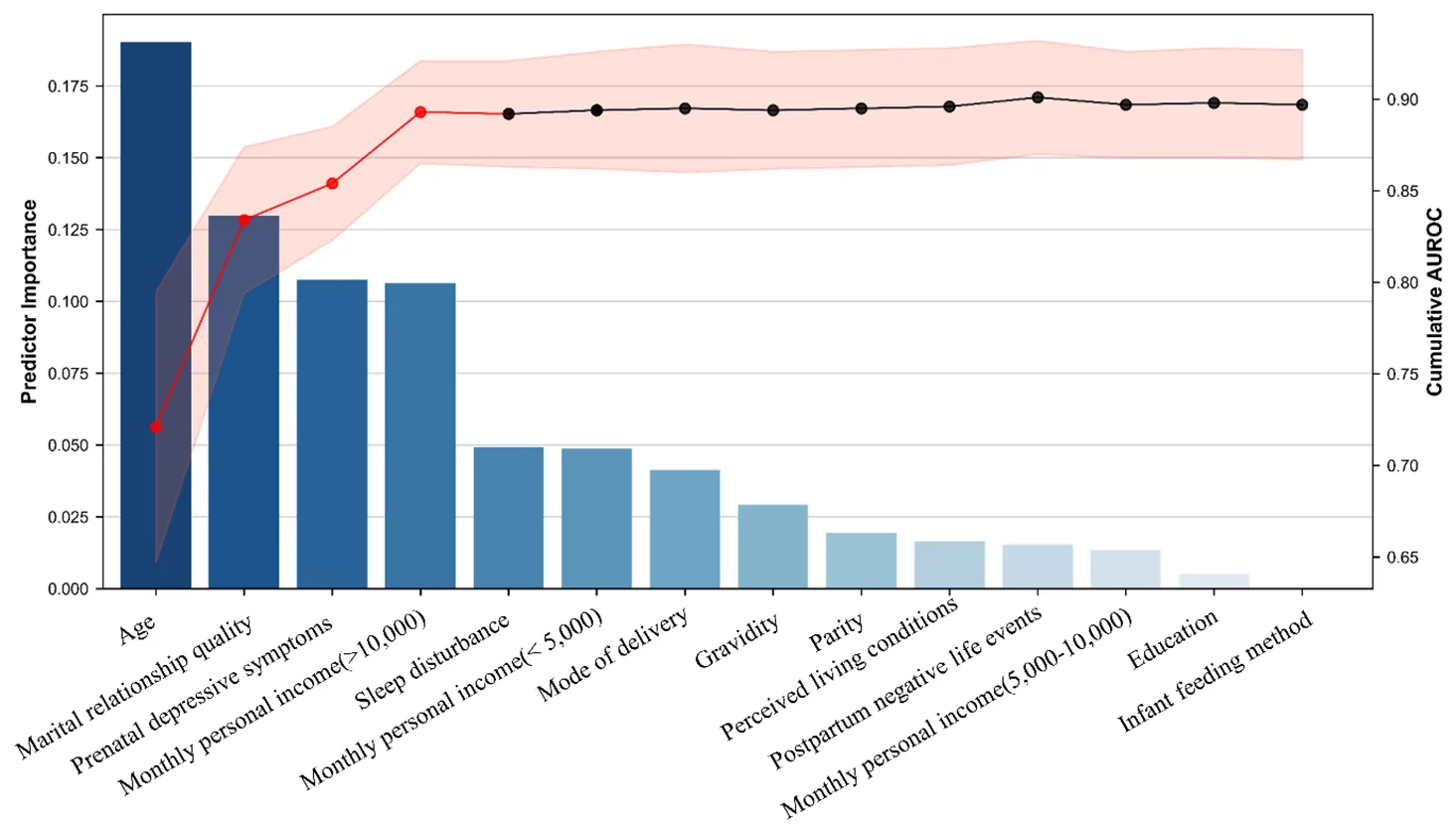
Comparison of the performance derived from XGBoost model constructed with various variable combinations based on variable importance. Bars show the relative importance of candidate predictors in the XGBoost model, and the line shows the cumulative AUROC after sequentially adding predictors according to importance ranking. Red points indicate the simplified predictor set used for the web-based calculator. AUROC, area under the receiver operating characteristic curve; XGBoost, eXtreme Gradient Boosting.

A four-predictor subset comprising age, marital relationship quality, prenatal depressive symptoms, and monthly personal income provided a favorable balance between discrimination and model parsimony. Based on this simplified predictor set, a web-based calculator (**Figure 7**) was developed to facilitate individualized risk estimation of screening-positive postpartum depressive symptoms (https://gt-ustc.shinyapps.io/postpartum_depression/).

**Figure 7:**
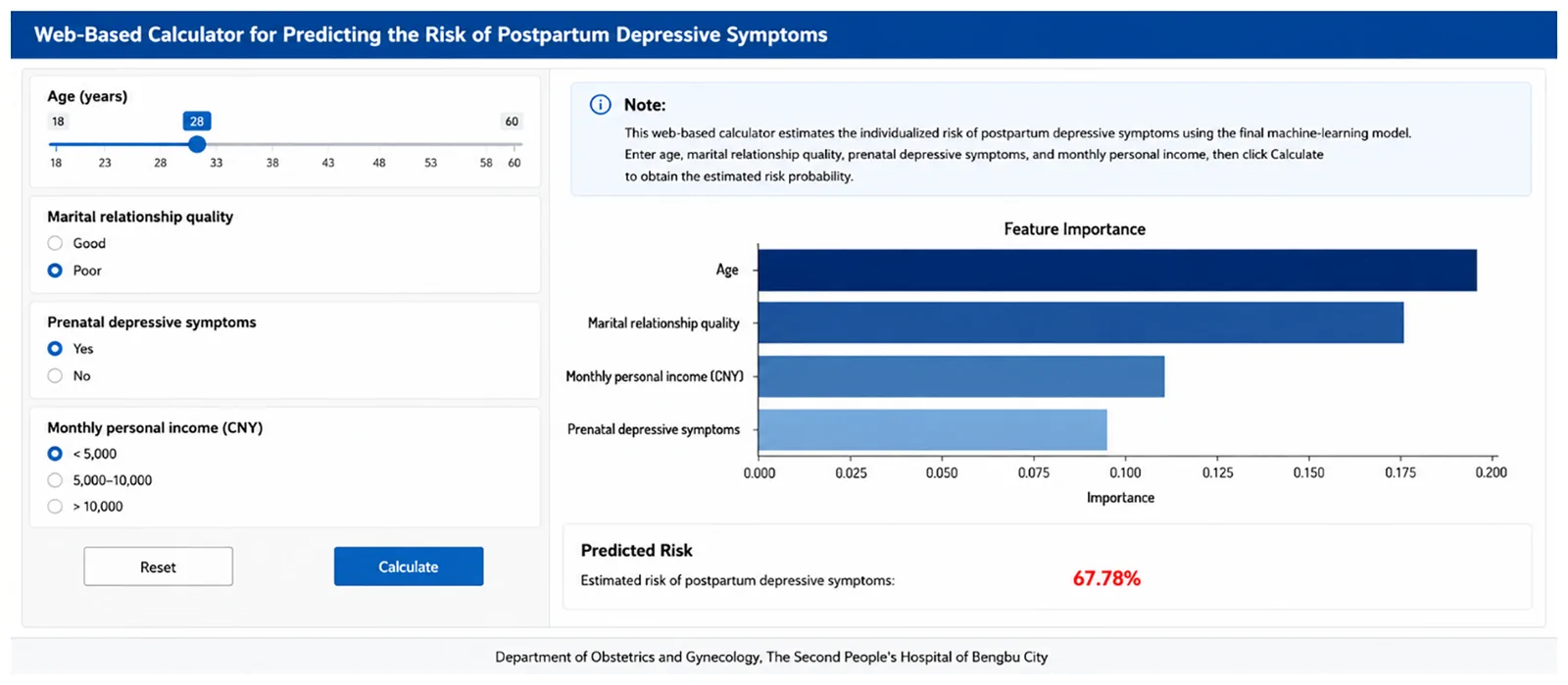
Web-based calculator for individualized risk estimation of postpartum depressive symptoms. The calculator was developed using the simplified predictor set derived from the final XGBoost model, including age, marital relationship quality, prenatal depressive symptoms, and monthly personal income. Users can enter individual predictor values to obtain an estimated risk probability. EPDS, Edinburgh Postnatal Depression Scale.

## 4 Discussion

### 4.1 Principal findings

In this dual-cohort study, we developed and externally validated machine learning models for predicting postpartum depressive symptoms at 6 weeks postpartum. XGBoost was selected for subsequent analyses and achieved AUCs of 0.845 in the internal test set and 0.820 in the external validation cohort. SHAP analysis indicated that age, marital relationship quality, prenatal depressive symptoms, monthly personal income, and sleep disturbance were important model-based contributors. A web-based calculator was developed using a simplified predictor set to support individualized risk estimation. Collectively, these findings provide preliminary external support for the model and suggest potential value for risk stratification in perinatal care, although further validation is required to establish broader generalizability.

### 4.2 Methodological implications

Regarding model development, XGBoost achieved the numerically highest AUC and showed well-balanced performance among the evaluated algorithms. This finding is broadly consistent with previous postpartum depression prediction studies, which have reported AUC values ranging from 0.70 to 0.90 (36). For example, Hochman et al. developed an XGBoost-based model using Israeli electronic health records to predict 1-year postpartum depression, achieving an AUC of 0.712 (25). Similarly, Huang et al. reported favorable performance of XGBoost compared with several other machine learning algorithms (29). Methodologically, its ability to capture nonlinear relationships and predictor interactions while incorporating regularization may partly explain its performance in multidimensional clinical data (37, 38). However, the differences among XGBoost, LightGBM, and random forest were small, and no formal pairwise statistical comparisons were performed. Therefore, the selection of XGBoost should be interpreted as reflecting a slight numerical advantage rather than statistically established superiority. Beyond discrimination, calibration was generally acceptable in the internal test set, whereas the negative calibration intercept indicated overestimation of absolute risk in the external validation cohort. This may partly reflect differences in outcome prevalence and population characteristics and suggests that recalibration may be needed when the model is applied to new populations (39, 40). Decision curve analysis further indicated potential net clinical benefit relative to the treat-all and treat-none strategies across a range of threshold probabilities. Together, these findings support further evaluation of the model for risk stratification while highlighting the need for additional validation and recalibration before broader clinical implementation.

Sample size adequacy and validation rigor are important methodological considerations in prediction model development. In the development cohort, 153 EPDS-positive events were observed among 403 women, and 16 raw candidate predictors were considered before feature selection, providing a reasonable basis for exploratory model development (41). Nevertheless, one-hot encoding of categorical variables increases the number of input features, thereby increasing model dimensionality, while machine learning models may remain susceptible to overfitting in modest-sized datasets (42). To reduce this risk, we applied recursive feature elimination, five-fold cross-validation for hyperparameter tuning, and regularized ensemble modeling (43). Furthermore, the dual-cohort design enabled independent external validation, in which the XGBoost model achieved acceptable discriminative performance with an AUC of 0.820. External validation is recommended to assess model reproducibility and transportability, because performance estimates based only on internal validation may be overly optimistic. The external validation cohort was kept separate from all model development procedures, including feature selection, hyperparameter tuning, model training, model selection, and predictor simplification, thereby minimizing the risk of information leakage and providing a more independent assessment of model performance (44). However, both cohorts were recruited from the same regional healthcare context in China, and the external validation cohort had a relatively modest sample size with only 39 EPDS-positive events (45). Therefore, these findings should be interpreted cautiously, and further validation in larger, prospective, multicenter cohorts across diverse regions and healthcare settings is needed to confirm the generalizability of the model.

In addition, the EPDS-based outcome definition should be considered when interpreting both the EPDS-positive rates and model performance. In the present study, the EPDS-positive rate was 38.0% in the development cohort and 26.0% in the external validation cohort, which is consistent with previous epidemiological studies (2, 46–49). Importantly, these estimates represent screening-positive rates rather than the diagnostic prevalence of clinically confirmed postpartum depression. The EPDS cut-off of ≥9 was adopted to prioritize early risk identification and reduce missed cases, consistent with previous EPDS validation studies (50). Individual participant data meta-analyses have shown that lower EPDS thresholds generally increase sensitivity, whereas higher thresholds improve specificity at the expense of sensitivity. For example, pooled sensitivity decreased from 0.85 at ≥10 to 0.66 at ≥13, while specificity increased from 0.84 to 0.95 (51). Consistently, prevalence estimates varied from 27.8% using EPDS ≥9 to 9.0% using EPDS ≥14 (51). From a machine learning perspective, this relatively sensitive threshold may have improved class balance and supported early risk identification, but it may also influence threshold-dependent metrics such as sensitivity, specificity, precision, and F1 score. Overall, the present model should be interpreted as a screening and risk-stratification tool for postpartum depressive symptoms rather than a diagnostic model for clinically confirmed postpartum depression.

### 4.3 Clinical implications and model interpretation

SHAP analysis identified age, marital relationship quality, prenatal depressive symptoms, monthly personal income, and sleep disturbance as important contributors to model predictions. Younger age was associated with higher predicted risk within the model, consistent with previous evidence identifying younger maternal age as a relevant predictor of postpartum depressive symptoms (22, 52). However, elevated risk has also been reported among women older than 35 years, suggesting heterogeneity in age-related associations (52, 53). Poor marital relationship quality was associated with higher predicted risk, consistent with previous studies linking partner conflict, poor marital adjustment, and insufficient social support with postpartum depressive symptoms (54, 55). Prenatal depressive symptoms were another major contributor (25, 28, 52, 56), which may reflect the persistence of depressive trajectories from pregnancy into the postpartum period or increased vulnerability among women with previous or ongoing emotional symptoms (57). Lower monthly personal income was associated with higher predicted risk, consistent with meta-analytic evidence linking low socioeconomic status to postpartum depression or depressive symptoms (23, 58). Similarly, sleep disturbance was associated with higher predicted risk, in line with previous studies suggesting potential roles of impaired emotional regulation, serotonergic dysregulation, altered prefrontal control, and inflammatory activation in postpartum depressive symptoms (59–61). Importantly, these variables should be interpreted as predictors contributing to model output rather than causal determinants of postpartum depressive symptoms. Nevertheless, their clinical accessibility is an important advantage. Most of these predictors can be obtained from routine obstetric records or brief self-report assessments, making them feasible for early screening in perinatal care settings. In clinical practice, the model may help clinicians identify women who warrant closer follow-up, further psychological assessment, or timely referral for mental health support.

To facilitate clinical translation, we developed a web-based calculator based on a simplified predictor set. This tool provides an intuitive interface for individualized risk estimation and may support risk stratification during postpartum follow-up (62). However, the calculator should be regarded as a screening support tool rather than a diagnostic instrument, and women identified as high risk should receive further evaluation using validated screening scales or structured clinical assessment. Before routine implementation, the calculator requires prospective validation in broader populations and different healthcare settings. If implemented in future clinical settings, its discrimination and calibration should be periodically monitored, with recalibration, model updating and ongoing maintenance performed as needed to maintain reliability and clinical relevance.

### 4.4 Limitations and future directions

Several limitations of this study warrant careful consideration. First, the retrospective design and modest overall sample size may introduce selection bias and restrict causal inference. In addition, the exclusion of participants with substantially incomplete data may have affected the representativeness of the study sample and model performance estimates. Second, although an independent external validation cohort was included, both cohorts were recruited from hospitals within the same regional healthcare context in China, and the external validation cohort contained a limited number of EPDS-positive events. These factors may limit the precision of external performance estimates and restrict the broader generalizability of the model to other regions, healthcare systems, and populations. Third, the outcome was assessed using the self-reported EPDS without confirmation by structured clinical diagnostic interviews. Therefore, the model predicts EPDS-defined postpartum depressive symptoms rather than clinically confirmed postpartum depression. The use of an EPDS cut-off of ≥9 may increase sensitivity for early screening but reduce specificity compared with higher thresholds, which may introduce measurement bias and limit comparability with studies using different EPDS cut-offs. Fourth, reliance on self-reported psychosocial predictors may introduce recall and reporting bias. Additionally, collecting certain postpartum variables close to the 6-week outcome assessment may limit the model’s utility for early prediction during pregnancy or delivery. Moreover, objective biological markers, such as hormonal, inflammatory, or genetic indicators, were not incorporated. Finally, although a web-based calculator was developed, its prospective clinical impact on screening efficiency, clinical decision-making, and patient outcomes has not yet been evaluated. Future multicenter, prospective, and cross-cultural studies with larger and more diverse populations, structured diagnostic assessments, standardized psychosocial instruments, multimodal biological data, clinical impact evaluations, and planned model updating strategies are needed to further validate and improve the model.

## 5 Conclusion

This study developed and externally validated an explainable XGBoost model for predicting postpartum depressive symptoms. The selected model demonstrated promising discrimination in both the internal test set and the external validation cohort. SHAP analysis enhanced model interpretability by identifying important model-based contributors, including age, marital relationship quality, prenatal depressive symptoms, monthly personal income, and sleep disturbance. A web-based risk calculator was developed to support individualized risk assessment. Overall, this study provides an interpretable framework for early screening and risk stratification of postpartum depressive symptoms, although further prospective multicenter validation and clinical utility evaluation are needed before broader clinical implementation.

## Data Availability

The original contributions presented in the study are included in the article/**Supplementary Material**. Further inquiries can be directed to the corresponding authors.
