# Supplementary material for: Development and external validation of an explainable XGBoost model with a web-based calculator for risk prediction of postpartum depressive symptoms
Source: Front Psychiatry. 2026 Sep 15;17:1887667. doi: 10.3389/fpsyt.2026.1887667 (PMC13619944; doi:10.3389/fpsyt.2026.1887667)
Supplement: Supplementary file 1 [file SupplementaryFile1.docx]

**Supplementary Table S1** Hyperparameter search spaces and final selected hyperparameters for the five machine learning models

| Model | Hyperparameter | Search space | Final selected value |
| --- | --- | --- | --- |
| LR | C | 0.005, 0.01, 0.05, 0.06, 0.1, 0.5, 1, 10, 100 | 10 |
|  | penalty | l1, l2 | l1 |
|  | solver | liblinear, saga | liblinear |
|  | fit_intercept | True, False | True |
| SVM | C | 0.0001, 0.001, 0.01, 0.1, 1 | 0.1 |
|  | kernel | linear, rbf, poly, sigmoid | linear |
|  | gamma | scale, auto, 0.0001, 0.001, 0.01, 0.1 | N/A (linear kernel) |
| RF | n_estimators | 100–1000 (step = 100) | 100 |
|  | max_features | sqrt, log2, None | log2 |
|  | max_depth | None, 3, 5, 7, 9, 10, 11, 12, 13, 15 | None |
|  | criterion | gini, entropy | gini |
|  | min_samples_leaf | 3, 5, 7, 9, 11, 13, 15, 20, 30, 40, 50, 100 | 5 |
| XGBoost | learning_rate | 0.3, 0.1, 0.01, 0.001 | 0.01 |
|  | n_estimators | 100–1000 (step = 100) | 300 |
|  | max_depth | 3, 6, 9, 12, 15 | 12 |
|  | min_child_weight | 3, 6, 9, 12, 15 | 3 |
|  | gamma | 0.0–1.9 (step = 0.1) | 1.6 |
|  | colsample_bytree | 0.75–1.00 (step = 0.05) | 0.80 |
|  | subsample | 0.70–1.00 (step = 0.05) | 0.75 |
|  | reg_alpha | 0, 0.00001, 0.01, 0.1, 1, 100 | 0.01 |
| LightGBM | learning_rate | 0.1, 0.01, 0.001, 0.0001, 0.00001 | 0.01 |
|  | n_estimators | 100–1000 (step = 100) | 400 |
|  | max_depth | 3–30 (step = 3) | 3 |
|  | num_leaves | 10–100 (step = 10) | 80 |
|  | subsample | 0.70–1.00 (step = 0.05) | 0.85 |
|  | colsample_bytree | 0.70–1.00 (step = 0.05) | 0.85 |
|  | min_child_samples | 10, 13, 15, 16, 18, 19, 20, 21, 22 | 21 |
|  | min_child_weight | 0.001, 0.002 | 0.002 |
|  | subsample_freq | 0, 1, 2, 3, 4 | 1 |
|  | cat_smooth | 0, 10, 20 | 0 |

Note: LR, logistic regression; SVM, support vector machine; RF, random forest; LightGBM, Light Gradient Boosting Machine; XGBoost, eXtreme Gradient Boosting; N/A, not applicable. Hyperparameters were optimized using randomized search with five-fold stratified cross-validation, with ROC-AUC as the optimization metric and a fixed random seed of 417.

**SUPPLEMENTARY FIGURE S1** Confusion matrices of the final XGBoost model. (A) Held-out internal test set. (B) Independent external validation cohort. Binary classifications were generated using a fixed probability threshold of 0.50. XGBoost, eXtreme Gradient Boosting.
